# Wounding of *Arabidopsis halleri* leaves enhances cadmium accumulation that acts as a defense against herbivory

**DOI:** 10.1007/s10534-015-9829-9

**Published:** 2015-03-10

**Authors:** Sonia Plaza, Johann Weber, Simone Pajonk, Jérôme Thomas, Ina N. Talke, Maja Schellenberg, Sylvain Pradervand, Bo Burla, Markus Geisler, Enrico Martinoia, Ute Krämer

**Affiliations:** 1 Institute of Plant Biology, University of Zurich, 8008 Zurich, Switzerland; 2 Center for Integrative Genomics, University of Lausanne, 1015 Lausanne, Switzerland; 3 Department of Plant Physiology, Ruhr University Bochum, Universitaetsstrasse 150 ND3/30, 44801 Bochum, Germany; 4 Present Address: Institute of Plant Sciences, University of Bern, 3013 Bern, Switzerland; 5 Present Address: Max Planck Institute of Molecular Plant Physiology, 14476 Potsdam, Germany; 6 Present Address: University Hospital Zurich, University of Zurich, 8006 Zurich, Switzerland; 7 Present Address: Department of Biology, University of Fribourg, 1700 Fribourg, Switzerland

**Keywords:** Cadmium (Cd), Metal hyperaccumulator plant, Iron (Fe), Jasmonate, Insect herbivory, Pieris rapae, Chemical ecology, Elemental defence, Phytoremediation

## Abstract

Approximately 0.2 % of all angiosperms are classified as metal hyperaccumulators based on their extraordinarily high leaf metal contents, for example >1 % zinc, >0.1 % nickel or >0.01 % cadmium (Cd) in dry biomass. So far, metal hyperaccumulation has been considered to be a taxon-wide, constitutively expressed trait, the extent of which depends solely on available metal concentrations in the soil. Here we show that in the facultative metallophyte *Arabidopsis halleri*, both insect herbivory and mechanical wounding of leaves trigger an increase specifically in leaf Cd accumulation. Moreover, the Cd concentrations accumulated in leaves can serve as an elemental defense against herbivory by larvae of the Brassicaceae specialist small white (*Pieris rapae*), thus allowing the plant to take advantage of this non-essential trace element and toxin. Metal homeostasis genes are overrepresented in the systemic transcriptional response of roots to the wounding of leaves in *A. halleri*, supporting that leaf Cd accumulation is preceded by systemic signaling events. A similar, but quantitatively less pronounced transcriptional response was observed in *A. thaliana*, suggesting that the systemically regulated modulation of metal homeostasis in response to leaf wounding also occurs in non-hyperaccumulator plants. This is the first report of an environmental stimulus influencing metal hyperaccumulation.

**Electronic supplementary material:**

The online version of this article (doi:10.1007/s10534-015-9829-9) contains supplementary material, which is available to authorized users.

## Introduction

The essential micronutrients zinc and nickel, as well as the non-essential metal cadmium (Cd), can act as potent toxins when present in excess, endangering environmental and human health (Clemens et al. 2013). Within the characteristic vegetation shaped by high selection pressures occurring on heavy-metal rich soils, a small proportion of metallophyte plants—estimated to constitute around 1 % —accumulate extraordinarily high leaf metal concentrations (Baker and Brooks 1989). These rare metal hyperaccumulator plants are of considerable interest for the development of phytoremediation and phytomining technologies. To date, metal hyperaccumulation has been considered to be a constitutive trait, which is expressed taxon-wide under all conditions as long as bioavailable metal concentrations are sufficient in the soil. There is some evidence that the ecological role of metal hyperaccumulation in plants is to act as an elemental defense against pathogen attack and insect herbivory (Boyd 2007; Boyd and Martens 1992).

In the Brassicaceae family of flowering plants, *Arabidopsis halleri* is a well-known Zn hyperaccumulator that also exhibits hypertolerance to both Zn and Cd (Verbruggen et al. 2009). Moreover, *A. halleri* individuals collected from one natural population have been reported to contain hyperaccumulator concentrations of Cd exceeding 100 µg g^−1^ in leaf dry biomass (Dahmani-Muller et al. 2000), but Cd hyperaccumulation appeared not to be a species-wide trait (Krämer 2010). As a member of the taxonomic sister group and very close relative of the genetic model plant *A. thaliana*, *A. halleri* is gaining increasing attention in comparative genomics studies addressing the molecular basis of metal hyperaccumulation and hypertolerance (Krämer 2010; Verbruggen et al. 2009). Alongside genetic approaches, these studies identified candidate genes, which were then functionally characterized. The stable genetic transformation—so far uniquely used in *A. halleri* among all metal hyperaccumulator taxa—has proven invaluable in demonstrating the biological functions of candidate genes (Hanikenne et al. 2008).

Using transgenic *A. halleri Heavy Metal APTase4* (*HMA4*) RNA interference lines (Hanikenne et al. 2008), which are impeded in both Zn and Cd accumulation, Kazemi-Dinan et al. (2014) conducted a stringent test of the elemental defense hypothesis. Both wild-type and transgenic non-accumulating lines were grown under identical conditions prior to paired-choice experiments using the specialist chewing herbivorous insects *Athalia rosae* and *Phaedon cochleariae*. In these assays, herbivores exhibited preferred feeding on non-accumulating transgenic lines by comparison to the hyperaccumulating wild type, in agreement with the elemental defense hypothesis (Kazemi-Dinan et al. 2014).

In plants, leaf wounding through insect herbivory is well known to induce the biosynthesis of secondary metabolites which act as defenses against herbivory (Mithofer and Boland 2012). Among these defense compounds, constitutive and herbivory-inducible production of glucosinolates, for example, is characteristic of the Brassicaceae including also *A. halleri*. Research testing the elemental defense hypothesis has not so far examined whether biotic stress has an effect on the extent of metal accumulation in hyperaccumulator plants. Here we show that herbivory by larvae of the small white butterfly *Pieris rapae*, and mechanical wounding simulating herbivory, both enhance Cd accumulation in leaves of *A. halleri*. We demonstrate that the accumulated levels of Cd deter feeding by *Pieris rapae*. The analysis of the wounding-induced systemic transcriptome in roots suggests a pronounced remodeling of metal homeostasis in response to wounding, which is also occurring—to a lesser extent—in *A. thaliana*. Our data show that Cd hyperaccumulation is inducible in *A. halleri*. This has profound consequences for future field and laboratory studies of hyperaccumulator plants. Moreover, our results suggest that future work should address the roles of transition metals in defense responses of *A. thaliana*.

## Methods

### Plant growth and wounding

Hydroponically grown (Massonneau et al. 2001) 9-week-old *Arabidopsis halleri* (accession Langelsheim, N51°56′35.2″, E10°20′56.3″) and *A. thaliana* (where appropriate) were transferred into a medium supplemented with 0.5 μM CdCl_2_ for 5 days before the initiation of wounding by herbivory through *Pieris rapae* larvae for 24 h, or mechanical wounding carried out with a razor blade on a single leaf simulating extent and shape of herbivory by *P. rapae*. Roots were harvested for transcript profiling 5 h after mechanical leaf wounding, or the remaining intact leaves were harvested 72 h after the initiation of wounding for the quantification of Cd concentrations by AAS (Bovet et al. 2003). Leaf biomass eaten was quantified according to leaf surface areas scanned before and after feeding of an insect for 3 h on detached leaves from plants exposed to 0.5 μM CdCl_2_ or no Cd (controls) for 5 days (Leica IM1000 software). Seven-month-old *A. halleri* cultivated in pots (60 mm ∅) of a 1:1 (v/v) mixture of vermiculite and an autoclaved, sieved (5 mm) metal-contaminated native soil (Langelsheim; exchangeable Cd 13.3 mg kg^−1^) were mechanically wounded simulating herbivory four times at 72-h intervals. For each wounding event, an undamaged leaf was perforated multiple times with a 1-mL plastic pipette tip by pressing against a fragment of a lid of a plastic petri dish. Whole shoots were harvested 72 h after the fourth wounding event and rinsed in ultrapure water. Damaged leaves and leaves that had been in direct physical contact with the soil were removed from each plant. The remaining portion of each plant was dried and acid-digested before element analysis by ICP-OES (Becher et al. 2004). Statistics were done using SPSS 13. For the determination of exchangeable concentrations of elements in soil, 1 g soil, air-dried and sieved through a 2-mm mesh, was mixed with 10 mL 1 M ammonium acetate, pH 7.0, in a 15-mL screw-capped polypropylene tube, then shaken horizontally overnight at 150 rpm at room temperature, followed by filtering through Whatman paper no. 1. Before analysis by ICP-OES, 1 mL of 65 % nitric acid was added to each sample.

### RNA extraction and microarrays

Total root RNA was extracted with the RNeasy midi kit (Qiagen); mRNA was amplified with the MessageAmp aRNA II kit (Ambion). Five µg of aRNA were reverse transcribed into cyanin3- or cyanin5-labeled cDNA, and hybridized onto microarrays (GEO accession number GPL6147) containing 25,000 gene-specific tags for the *A. thaliana* genome (Hilson et al. 2004). Within-species changes in transcript levels between control and wounded plants were analyzed by two color co-hybridization of the labeled cDNAs. After print tip lowess normalization (Yang et al. 2002) of raw data and statistical data analysis with the LIMMA package (http://www.bioconductor.org/), genes with an expression fold change ≥1.5 and a *P* value <0.05 (moderate *t* statistics, four biological replicates) were considered as significantly differentially expressed between non-wounded control and wounded plants. Among regulated genes, an overrepresentation of metal homeostasis genes (Talke et al. 2006), genes upregulated under iron deficiency in roots (Colangelo and Guerinot 2004), and genes regulated by 12-oxo-phytodienoic acid (OPDA) (Taki et al. 2005), cold, drought and UV-B (abiotic stress) (Kilian et al. 2007), herbivory (Reymond et al. 2004), methyl jasmonate (Nemhauser et al. 2006) or NaCl (Gong et al. 2005) was assessed using Fisher’s exact test.

## Results

**Fig. 1:**
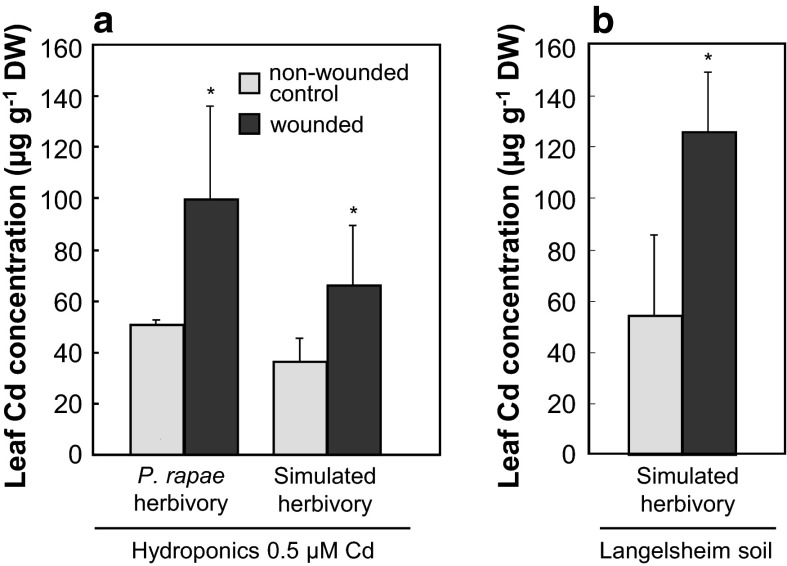
Cadmium hyperaccumulation in *Arabidopsis halleri* is induced by herbivory and mechanical leaf wounding. **a** Leaf Cd concentrations were determined 72 h after wounding of *A. halleri* by herbivory through larvae of *Pieris rapae* or by mechanical wounding simulating herbivory. Plants were cultivated hydroponically. **b** Leaf Cd concentrations were determined upon mechanical wounding simulating herbivory in *A. halleri* cultivated on its native heavy metal-contaminated soil. Wounding was carried out four times consecutively at 72-h intervals, each time removing a total of approximately 50 % of the area of one single leaf in between secondary veins on both sides of the mid-rib, with harvest of entire shoots 72 h after the last wounding event (color-coding of *bars* as in 1a). Shown data are arithmetic mean ± SD of *n* = 3 to 5 replicate plants from one experiment representative of two to three independent experiments. *Asterisks* indicate significant differences (*P* < 0.05) between wounded and non-wounded plants (Mann–Whitney U test). *DW* dry biomass

To investigate the relationship between insect herbivory and Cd accumulation in *A. halleri*, larvae of *Pieris rapae* were allowed to feed for 24 h on 9-week-old *A. halleri* and *A.* *thaliana* plants, 5 days after the addition of a non-toxic concentration of 0.5 µM Cd to the hydroponic plant growth medium. 72 h after the initiation of feeding, intact leaves of *A. halleri* subjected to herbivory contained 1.97- to 3.72-fold higher Cd concentrations than leaves of non-wounded control plants (Fig. 1a; *P* = 0.02). By contrast, we did not observe significant effects of wounding on leaf concentrations of Zn or other metals, and herbivory had no effect on leaf Cd concentrations in *A. thaliana* (not shown). Mimicking herbivory by removing 50 % of one leaf blade from Cd-supplemented *A.* *halleri* plants using a razor blade resulted in a comparable increase in leaf Cd concentrations (*P* = 0.007) (Fig. 1a). This confirmed that *A. halleri* responds to leaf wounding by increasing the accumulation of Cd in leaves. We also determined Cd accumulation in *A. halleri* plants grown on a metal-contaminated soil collected at the site of origin of the *A. halleri* population (Langelsheim, Germany). Compared to non-wounded plants grown on this Cd-contaminated soil, leaves of mechanically wounded plants contained higher Cd concentrations (Fig. 1b), with the average concentration exceeding the threshold concentration of 100 µg g^−1^ Cd in leaf dry biomass, which is used to identify Cd hyperaccumulators in the field (Baker and Brooks 1989).

**Fig. 2:**
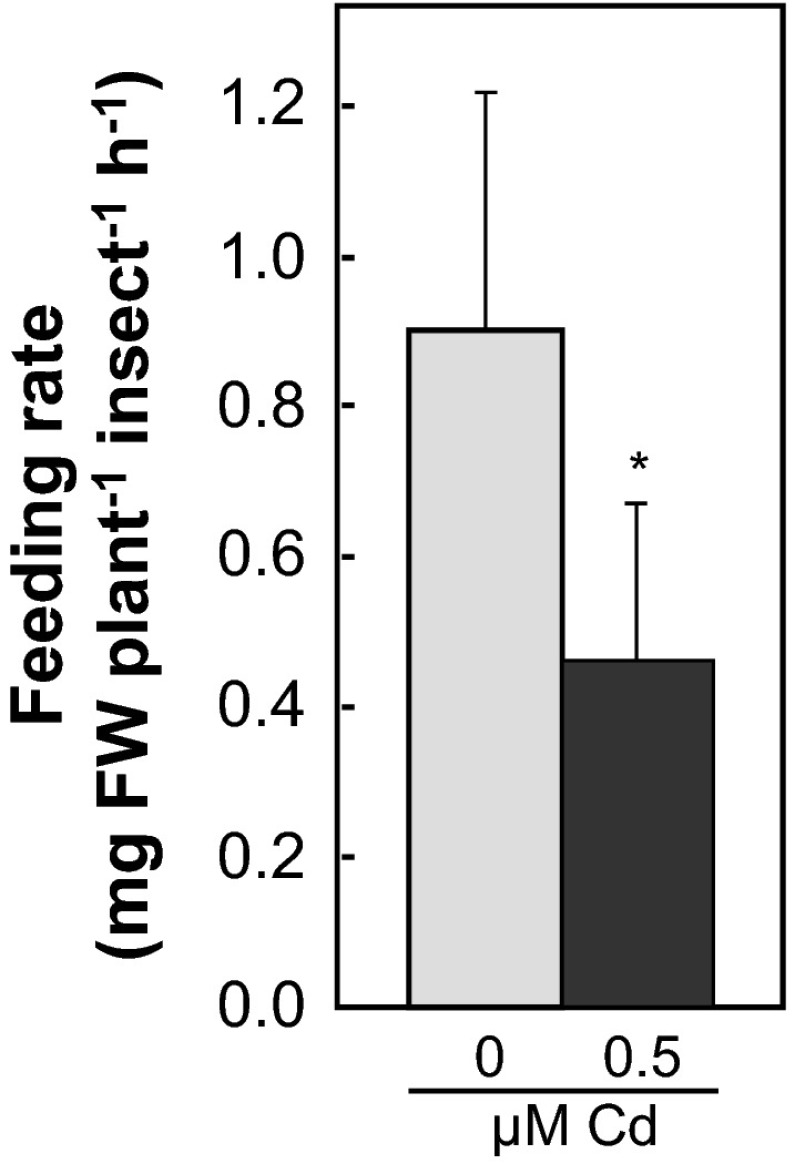
Insect herbivory is decreased when Cd is available for accumulation by *Arabidopsis halleri*. **a** The rate of consumption of *A. halleri* leaf biomass by larvae of *Pieris rapae* was quantified 5 days after supplementation of hydroponic solution with no or 0.5 µM CdCl_2_. Shown data are arithmetic mean ± SD of *n* = 3 to 5 replicate plants from one experiment representative of two to three independent experiments. *Asterisks* indicate significant differences (*P* < 0.05) between wounded and non-wounded plants (Mann–Whitney U test). *FW* fresh biomass

From *A. halleri* cultivated in a Cd-supplemented hydroponic medium (see Fig. 1a above), larvae of *Pieris rapae* ate 49.0 ± 24.0 % (arithmetic mean ± SD; *P* = 0.032) less leaf biomass than from *A. halleri* cultivated without added cadmium (Fig. 2). 5 days later, the larvae fed on Cd-supplemented plants retained 3.26 ± 0.37 fold higher Cd concentrations (arithmetic mean ± SD; *P* = 0.029; 2.15 ± 0.22 and 0.66 ± 0.06 µg Cd g^−1^ dry biomass, respectively; data not shown) than larvae fed on plants grown in a medium without added Cd. This demonstrated that Cd accumulation in *A. halleri* can effectively deter insect herbivory.

**Fig. 3:**
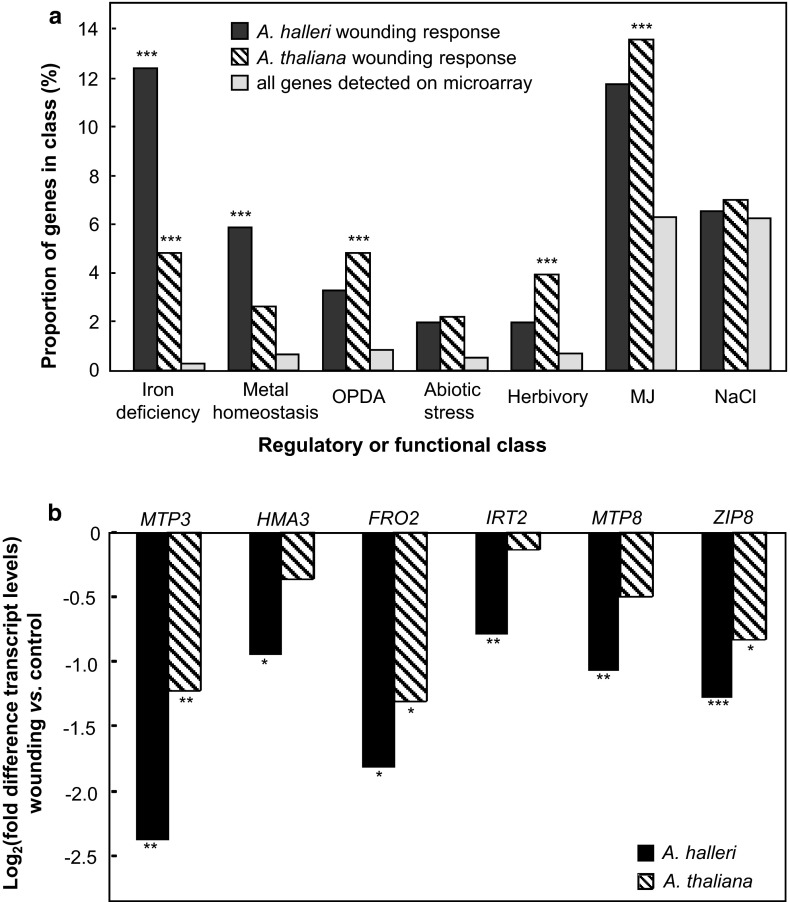
Comparison of systemic transcriptional responses of roots to simulated herbivory in *A. halleri* and *A. thaliana.* **a** Enrichment analysis. For each functional/regulatory class, *bars* represent the proportion of wounding-responsive genes contained therein, and the proportion of all genes detected on the CATMA arrays as a reference (***P < 0.001; Fisher’s exact test). **b** Average Log_2_ (fold changes) in root transcript levels of wounded versus non-wounded plants for selected iron deficiency response genes. Systemic wounding-induced changes in gene expression were determined in roots 5 h after leaf wounding relative to non-wounded controls using CATMA microarrays, and averaged from four independent experiments. Oxylipin family compounds: OPDA (12-oxo phytodienoic acid), MJ (methyl jasmonate)

Transpiration rates did not increase in response to wounding (data not shown). Therefore, wounding-induced Cd hyperaccumulation in *A. halleri* was hypothesized to involve increased Cd flux into the xylem of roots for transport into leaves via the transpiration stream. To investigate the systemic transcriptional response of roots to the wounding of leaves, we conducted comparative transcript profiling in roots of *A. halleri* and *A. thaliana* using microarrays harboring 25,000 gene-specific tags for the *A. thaliana* genome. Out of a total of 21,483 genes, for which expression signals were detected in *A. halleri*, the expression of 153 genes was more than 1.5-fold up- or downregulated in response to wounding (*P* < 0.05; 44 up, 109 down; Supplementary Table 1). In *A. thaliana*, the expression of 228 genes out of a total of 21,489 expressed genes changed in response to wounding (*P* < 0.05; 44 up, 184 down; Supplementary Table 2). The classification of wounding-responsive genes of *A. halleri* according to functional annotations (Usadel et al. 2005) and published responses (Colangelo and Guerinot 2004; Gong et al. 2005; Kilian et al. 2007; Nemhauser et al. 2006; Reymond et al. 2004; Taki et al. 2005) indicated that Fe-deficiency response genes (Colangelo and Guerinot 2004), metal homeostasis genes (Talke et al. 2006) and 12-oxo-phytodienoic acid (OPDA)-responsive genes (Taki et al. 2005) were 44-, 8.3- and 3.9-fold overrepresented, respectively, compared to the representation of these classes of genes among all expressed genes (Fig. 3a). By comparison, among systemically wounding-responsive genes of *A.* *thaliana*, there was no statistically significant overrepresentation of metal homeostasis genes in general, but iron deficiency response genes were 17-fold and OPDA-responsive genes were 5.8-fold overrepresented, respectively.

*Metal Tolerance Protein3* (*MTP3*), which encodes a vacuolar membrane Zn^2+^/H^+^ antiporter in *A. thaliana* (Arrivault et al. 2006), was the most strongly regulated transcript in roots in response to leaf wounding in *A. halleri*, with a decrease by 81 % compared to non-wounded controls (Fig. 3b; see also Supplementary Table 1). The response of *MTP3* transcript levels was similar, but quantitatively less pronounced, in *A. thaliana*, with a decrease by 57 % compared to non-wounded controls. Upon wounding of *A. halleri*, a decrease in transcript levels was also observed for *Heavy Metal ATPase3* (*HMA3*), which encodes a putative vacuolar membrane Zn^2+^/Cd^2+^/Pb^2+^ pump (Gravot et al. 2004; Morel et al. 2009). Both *MTP3* and *HMA3* are part of the *Fe*-*deficiency Induced Transcription Factor1* (*FIT1*) regulon of *A. thaliana* (Colangelo and Guerinot 2004), i.e. their transcription is enhanced in roots under Fe deficiency dependent on the transcription factor FIT1. Indeed, transcript levels of *Ferric Reductase Oxidase2* (*FRO2*), *Iron*-*Regulated Transporter2* (*IRT2*) and *Metal Tolerance Protein8* (*MTP8*), all of which belong to the *FIT1* regulon, were also decreased in roots of wounded plants, again pronouncedly in *A. halleri* and not or only moderately in *A. thaliana* (Fig. 3b), among others (Supplementary Tables 1 and 2). The systemic wounding response of metal homeostasis genes neither encompassed the entire *FIT1* regulon, nor was it restricted to the *FIT1* regulon alone, including also, for example, the *Zinc*-*regulated transporter, Iron*-*regulated transporter Protein8* (*ZIP8*), which does not belong to the *FIT1* regulon (Fig. 3b). Using qPCR, the microarray data were confirmed for all these genes (Figure S1). Taken together, these results show that leaf wounding elicits a systemic transcriptional response in roots, in which metal homeostasis and Fe deficiency response gene transcript levels are suppressed more strongly in *A. halleri* than in *A. thaliana*.

## Discussion

To a considerable degree, research on metal hyperaccumulation was based on the implicit assumption that metal hyperaccumulation or hypertolerance mechanisms must be inducible by exposure of a hyperaccumulator plant to elevated concentrations of the cognate metal. By contrast, it came as a surprise that all candidate genes—including *HMA4*, *Metal Tolerance Protein1* (*MTP1*) and *Nicotianamine Synthase2* (*NAS2*) that are presently known to be of central functional importance—are constitutively highly expressed in *A. halleri* by comparison to closely related non-accumulators (Becher et al. 2004; Weber et al. 2004). A subgroup of highly expressed candidate genes of *A. halleri* consists of Zn deficiency response genes (Talke et al. 2006). Their transcript levels are generally high in *A. halleri* as a consequence of *HMA4*-mediated Zn depletion in roots (Hanikenne et al. 2008), but they also retain their responsiveness to Zn-mediated repression of transcript levels known for their homologues in *A. thaliana*. In agreement with this, root-shoot Zn partitioning in *A. halleri* depends on external Zn supply (Talke et al. 2006).

As shown here, both herbivory and mechanical leaf wounding enhanced leaf Cd accumulation in *A. halleri* (Fig. 1). This must involve altered activities of processes in roots, and thus systemic signaling. Wounding-triggered leaf-to-leaf systemic signaling was recently shown to involve the movement of electrical surface potential changes dependent on ionotropic glutamate receptor-related plant proteins and to occur very fast (Mousavi et al. 2013). It is surprising that in *A. halleri*, wounding-activated processes act preferentially or even specifically to enhance shoot Cd accumulation. Cd^2+^ is not an essential nutrient in higher plants so that it generally accumulates in plants along pathways of chemically related nutrient metal cations such as Fe^2+^ or Zn^2+^ (Clemens et al. 2013). Our data suggested a comparably large variation of leaf Cd accumulation (see Fig. 1). We attribute this to the difficulty of both administering reproducible degrees of wounding and preventing accidental wounding in control plants. Moreover, both insect herbivory and pathogens can trigger overlapping signaling pathways, for example those involving the oxylipin family of plant hormones including jasmonates. *A. halleri* is particularly prone to pathogen and insect pests, and thus it is technically difficult to entirely exclude the presence of all biotic stress in experiments (Maja Schellenberg, Ina Talke, Ricardo Stein, Enrico Martinoia and Ute Krämer, unpublished observations). We expect that, in addition to leaf wounding, other biotic factors also act to enhance leaf Cd accumulation. For example, it has been reported that the natural root microbiome of *A. halleri* had a modestly enhancing effect on leaf Cd accumulation (Farinati et al. 2009; Muehe et al. 2015), but also this system remains challenging to control (Farinati et al. 2011). Rich microbiomes are known to colonize both above- and below-ground organs of plants, adding to the complexity of these experiments (Bulgarelli et al. 2012; Horton et al. 2014; Lundberg et al. 2012).

Leaf wounding is known to activate oxylipin-based signaling (Nemhauser et al. 2006; Reymond et al. 2004; Taki et al. 2005). This was observed here in the systemic transcriptional response of roots of *A. thaliana* and—to a lesser degree—*A. halleri*, with the activation of transcriptional methyl jasmonate, 12-oxophytodienoic acid (OPDA) and herbivory responses (Fig. 3a). Especially among the responses of *A. halleri*, we observed a striking overrepresentation of Fe deficiency responses and metal homeostasis genes. Upon closer investigation, this response consisted to a large extent of the transcriptional repression of Fe deficiency response genes, in particular genes of the *FIT1* regulon (Supplementary Tables 1 and 2). Future studies will address whether or not this transcriptional response contributes to the wounding-induced leaf Cd accumulation response in *A. halleri*.

In *A. thaliana*, there was no increase in leaf Cd accumulation in response to wounding (data not shown). In agreement with this, despite a strong activation of herbivory responses in *A. thaliana* (see Fig. 3a), the transcriptional repression of Fe deficiency response genes was quantitatively far less pronounced in *A. thaliana*, with fewer genes detected to respond in our microarray analysis. Our data are consistent with a report that jasmonate treatment of *A. thaliana* resulted in decreased transcript levels of Fe deficiency response genes (Maurer et al. 2011).

Our transcript profiling identified some candidate genes for contributions to wounding-enhanced leaf Cd accumulation in *A. halleri*. *MTP3* was the most strongly repressed transcript in response to leaf wounding. In *A. thaliana*, transcription of the FIT1 target *MTP3* is activated when root Zn^2+^ uptake rates are enhanced under Fe deficiency and excess Zn. Under these conditions, the vacuolar-membrane localized MTP3 protein acts to sequester Zn^2+^ in vacuoles of root epidermal and cortex cells, thus decreasing shoot Zn accumulation (Arrivault et al. 2006). A decrease in *MTP3* expression would thus be predicted to enhance root-to-shoot Zn transport. *A. thaliana* MTP3 was found not to transport Cd^2+^, but the specificity of *A. halleri* MTP3 remains to be investigated. Another interesting candidate gene is the FIT1 target *HMA3*. Similar to MTP3, this P_1B_-type ATPase can also mediate the vacuolar sequestration of Zn^2+^, as well as of Cd^2+^ and Pb^2+^, in the root (Morel et al. 2009). The transcriptional repression of *HMA3* in roots of wounded *A. halleri* plants could thus decrease Cd immobilization inside roots and enhance the translocation of Cd into the shoots.

In *A. thaliana*, the systemic transcriptional repression of other FIT1 targets, for example *FRO2* (Fig. 3b) and *IRT1* (Supplementary Fig. 1), in the root is predicted to decrease the reduction of Fe^III^ chelates to Fe^2+^ and root uptake rates of Fe^2+^. In addition, root uptake of Cd^2+^ is expected to decrease because this heavy metal cation is primarily taken up through the high-affinity Fe^2+^ uptake system IRT1 in *A. thaliana*. The same effect is predicted in *A. halleri* unless this species possesses another, yet unidentified, root uptake system for Cd^2+^. If, indeed, *A. halleri* possessed such a root uptake system for Cd^2+^, the decreased expression of FIT1 regulon genes upon leaf wounding would have entirely different consequences for overall metal homeostasis: For example, the decreased expression of *FRO2*, in particular, would be expected to lower the concentration of extracellular Fe^2+^ competing with Cd^2+^ for uptake into root cells and to enhance plant Cd accumulation, and the latter was observed here in *A. halleri*. Future work will address each of these hypotheses.

In conclusion, our data suggest that in the metal hyperaccumulator *A. halleri* wounding induces signals that act systemically in the root to trigger enhanced leaf Cd accumulation, which in turn functions as a defense against attack by herbivores (Fig. 2), and possibly also pathogens (Boyd 2007). The existence of inducible metal hyperaccumulation in *A. halleri* provides strong circumstantial support for the elemental defense hypothesis (Boyd 2007). Furthermore, this observation requires an analysis of the underlying molecular mechanisms, and it will guide the design of future experiments addressing metal hyperaccumulation.

## Electronic supplementary material

Below is the link to the electronic supplementary material.

Supplementary material 1 (PDF 21 kb)

Supplementary material 2 (PDF 29 kb)

Supplementary material 3 (PDF 33 kb)
