# Supplementary material for: Wounding of Arabidopsis halleri leaves enhances cadmium accumulation that acts as a defense against herbivory
Source: Biometals. 2015 Mar 10;28(3):521–8. doi: 10.1007/s10534-015-9829-9 (PMC4427619; doi:10.1007/s10534-015-9829-9)
Supplement: Supplementary file 1 — Supplementary material 1 (PDF 21 kb) [file 10534_2015_9829_MOESM1_ESM.pdf]

Supplementary Table 1. Genes differentially expressed in roots of *A. halleri* 5 h after leaf wounding.

Given are AGI number, the ratio of the microarray signal of wounded *vs*. non-wounded plants calculated from the arithmetic mean of log-transformed values, annotation, short gene name, and functional or regulatory class for selected relevant classes. Microarray expression signals of shown genes changed by > 1.5-fold (increase or decrease) in response to leaf wounding, compared to non-wounded controls ($P < 0.05$). Plants were transfered into a hydroponic culture solution supplemented with 0.5 µM Cd 5 d prior to simulated herbivory on a single leaf. Annotations are underlined for genes differentially expressed in response to wounding when compared to controls in both *A. halleri* and in *A. thaliana*. OPDA: 12-oxo-phytodienoic acid.

AGI Number	Signal ratio	*P* value	Annotation	Name	Class
At3g60420	2.93	0.0087	<u>unknown protein, PRIB5 domain, phosphoglycerate mutase domain</u>		OPDA response
At3g16450	2.08	0.0018	<u>jacalin lectin family protein</u>		
At1g64160	1.95	0.0067	disease resistance-responsive family protein / dirigent family protein		NaCl response/ Methyl jasmonate response
At5g22530	1.93	0.0022	unknown protein		
At5g43580	1.91	0.0261	putative serine-type endopeptidase inhibitor		NaCl response/ Methyl jasmonate response
At5g52710	1.90	0.0007	heavy-metal-associated domain-containing protein		Metal homeostasis
At4g36060	1.84	0.0258	basic helix-loop-helix (bHLH) family protein, putative transcription factor		
At1g52890	1.81	0.0275	no apical meristem (NAM) family protein 19, NAC domain containing, putative transcription factor	ANAC019	Local herbivory response/ Methyl jasmonate response
At1g72360	1.80	0.0032	ethylene-responsive element-binding family protein, putative transcription factor		
At3g24550	1.80	0.0478	proline extensin-like receptor kinase 1	PERK1	
At4g33420	1.79	0.0004	<u>peroxidase 47 precursor, putative peroxidase, haem peroxidase domain</u>	PER47	Methyl jasmonate response
At2g16500	1.78	0.0340	arginine decarboxylase 1, polyamine biosynthesis	SPE1/ ARGDC/ ADC1	
At5g06730	1.73	0.0346	peroxidase 54 precursor, putative peroxidase, haem peroxidase domain	PER54	
At2g43520	1.70	0.0016	<u>trypsin inhibitor protein 2, putative trypsin inhibitor</u>	TI2	Methyl jasmonate response
At3g11930	1.69	0.0072	universal stress protein (USP) family protein		
At5g40000	1.67	0.0209	AAA-type ATPase family protein		NaCl response
At1g71450	1.65	0.0274	putative AP2 domain-containing transcription factor		
At2g36120	1.65	0.0194	pseudogene, glycine-rich protein		
At3g12145	1.64	0.0230	putative polygalacturonase inhibitor/ leucine-rich repeat protein	FLR1	
At3g49530	1.63	0.0042	no apical meristem (NAM) family protein 62, NAC domain containing, putative transcription factor	ANAC062	NaCl response
At3g57530	1.62	0.0217	calcium-dependent protein kinase 32	CPK32	
At5g61660	1.62	0.0470	glycine-rich protein		NaCl response
At5g61600	1.61	0.0146	ethylene-responsive element-binding family protein, putative transcription factor		Cold-, drought- and UVB-stress response
At3g23250	1.59	0.0397	myb domain protein 15, myb family transcription factor	MYB15	Methyl jasmonate response
At1g18300	1.58	0.0365	NUDIX hydrolase homolog 4, MutT-like protein	NUDT4	
At5g51440	1.57	0.0022	23.5 kDa mitochondrial small heat shock protein	HSP23.5-M	OPDA response
At5g47220	1.57	0.0416	ethylene-responsive element-binding factor 2, transcription factor	ERF2	Cold-, drought- and UVB-stress response/ Methyl jasmonate response
At3g23820	1.56	0.0068	<u>UDP-D-Glucuronate 4-epimerase 6, similar to NAD-dependent epimerase/dehydratase family protein</u>	GAE6	
At1g70230	1.55	0.0012	unknown protein		NaCl response/Methyl jasmonate response
At1g61660	1.55	0.0052	basic helix-loop-helix (bHLH) family protein, putative transciption factor		
At1g34360	1.55	0.0160	translation initiation factor 3 (IF-3) family protein		
At1g61740	1.55	0.0012	unknown protein		
At1g21910	1.55	0.0364	AP2 domain-containing transcription factor family protein, DREB subfamily of Ap2/EREBP protein family		Methyl jasmonate response
At4g38400	1.55	0.0020	expansin-like family protein 2	EXLA2	NaCl response
At5g49520	1.54	0.0359	WRKY family transcription factor 48	WRKY48	
At2g47190	1.54	0.0241	myb domain protein 2, myb family transcription factor	MYB2	
At5g22520	1.54	0.0040	unknown protein		
At1g59740	1.53	0.0042	proton-dependent oligopeptide transport (POT) family protein		

AGI Number	Signal ratio	*P* value	Annotation	Name	Class
At2g37130	1.53	0.0052	peroxidase 21 precursor, putative peroxidase, haem peroxidase domain	PER21	
At2g47400	1.52	0.0030	CP12 domain-containing protein, chloroplast stroma	CP12-1	
At2g39650	1.52	0.0364	unknown protein		
At1g72940	1.51	0.0057	putative disease resistance protein (TIR-NBS class)		Methyl jasmonate response
At4g00890	1.50	0.0354	proline-rich family protein, putative glycosyl hydrolase family 10 protein		
At1g13340	1.50	0.0085	unknown protein		
At2g40095	0.67	0.0163	unknown protein, contains alpha/beta hydrolase-related domain		
At5g40030	0.67	0.0190	putative serine/threonin protein kinase		
At1g80760	0.66	0.0023	major intrinsic protein (MIP) family, putative water channel, NOD26-like intrinsic protein 6;1	NIP6;1	
At1g68500	0.66	0.0232	unknown protein		
At5g52190	0.66	0.0309	sugar isomerase (SIS) domain-containing protein		
At5g59520	0.66	0.0141	ZRT-, IRT-related protein 2, ZIP family zinc transporter	ZIP2	Metal homeostasis
At3g51540	0.66	0.0406	unknown protein		
At1g54120	0.66	0.0124	unknown protein		Methyl jasmonate response
At4g17215	0.66	0.0009	unknown protein		
At1g47480	0.66	0.0327	unknown protein, similar to CXE carboxylesterase		
At2g42710	0.66	0.0100	ribosomal protein L1 family protein		
At1g68620	0.66	0.0165	unknown protein, similar to CXE carboxylesterase		
At1g18140	0.66	0.0125	laccase 1, putative laccase, multicopper oxidase, diphenol oxidase family protein	LAC1	
At4g24790	0.66	0.0234	putative DNA-directed DNA polymerase		
At5g06490	0.66	0.0018	zinc finger (C3HC4-type RING finger) family protein	L5D	Upregulated under iron deficiency
At1g03700	0.66	0.0001	integral membrane family protein		
At3g13610	0.66	0.0036	oxidoreductase, 2OG-Fe(II) oxygenase family protein		Upregulated under iron deficiency
At3g48450	0.66	0.0010	putative nitrate-responsive NOI protein		
At5g02050	0.65	0.0085	mitochondrial glycoprotein family protein / MAM33 family protein		
At1g10170	0.65	0.0082	NF-X1 type zinc finger family protein		
At3g45253	0.65	0.0083	non-LTR retrotransposon family (LINE)		
At2g40320	0.65	0.0006	unknown protein, similar to steroid hormone receptor/ transcription factor		
At1g43020	0.65	0.0328	unknown protein, similar to putative ternary complex factor MIP1		
At3g13950	0.65	0.0049	unknown protein		
At4g30270	0.65	0.0261	endo-xyloglucan transferase / xyloglucan endo-1,4-beta-D-glucanase	MERI5B/ MERI-5	Local herbivory response/ Methyl jasmonate response
At5g42655	0.65	0.0114	unknown protein, similar to disease resistance-responsive family protein, nucleoporin-related domain		
At4g38340	0.65	0.0068	RWP-RK domain-containing protein		
At1g70310	0.64	0.0078	spermidine synthase 2 (SPDSYN2) / putrescine aminopropyltransferase 2	SPDS2	
At2g33460	0.64	0.0326	Rop-interactive CRIB motif-containing protein 1, Cdc42/Rac-interactive binding (CRIB) motif, interacts with GTP-bound Rop1, possible role in microtubule organization	RIC1	
At2g31930	0.64	0.0278	unknown protein		
At5g05360	0.64	0.0194	unknown protein		
At3g02430	0.64	0.0339	unknown protein		
At5g02420	0.64	0.0011	unknown protein		
At5g02740	0.63	0.0178	unknown protein, nuleotide binding		NaCl response
At1g20330	0.63	0.0164	S-adenosyl-methionine-sterol-C-methyltransferase 2	SMT2	
At2g25680	0.63	0.0447	unknown protein, similar to sulfate transporter		
At2g29220	0.63	0.0145	putative lectin protein kinase		
At2g30395	0.63	0.0112	ovate family protein 17, unknown function	OFP17	
At1g74670	0.63	0.0136	putative gibberellin-responsive protein		Local herbivory response, NaCl response
At3g62830	0.63	0.0488	UDP-glucuronic acid decarboxylase 2, NAD-dependent epimerase/dehydratase family protein, synthesis of UDP-xylose	USX2	
At2g27370	0.63	0.0458	integral membrane family protein		

AGI Number	Signal ratio	*P* value	Annotation	Name	Class
At5g47200	0.63	0.0115	Arabidopsis Rab GTPase homolog D2b, GTP-binding protein	RABD2b/ Rab1A	
At4g38950	0.63	0.0173	kinesin motor family protein, similar to kinesin heavy chain		Upregulated under iron deficiency
At5g19970	0.63	0.0303	unknown protein		Methyl jasmonate response
At4g04460	0.63	0.0280	aspartyl protease family protein		
At3g01513	0.62	0.0167	unknown protein		
At3g21700	0.62	0.0043	unknown protein, similar to GTP-binding family protein, similar to Rab-type Ras small GTPase		
At1g09560	0.62	0.0391	germin-like protein 5, manganese ion binding, nutrient reservoir	GLP5	
At5g35935	0.62	0.0238	copia-like retrotransposon family		
At2g17130	0.62	0.0053	regulatory mitochondrial isocitrate dehydrogenase subunit 2 / NAD+ isocitrate dehydrogenase subunit 2	IDH2	
At3g07720	0.61	0.0044	kelch repeat-containing protein		Upregulated under iron deficiency
At2g35790	0.61	0.0138	unknown protein		
At1g78020	0.61	0.0340	senescence-associated protein-related		
At5g44460	0.61	0.0030	calcium-binding protein, putative		
At5g03570	0.61	0.0049	iron-regulated protein 2, vacuolar membrane Ni sequestering membrane transport protein	IREG2	Upregulated under iron deficiency/ Metal homeostasis
At5g22390	0.61	0.0007	unknown protein		
At4g33150	0.61	0.0134	lysine-ketoglutarate reductase/saccharopine dehydrogenase bifunctional enzyme	LKR/ SDH	
At4g02940	0.61	0.0056	oxidoreductase, 2OG-Fe(II) oxygenase family protein		Methyl jasmonate response
At4g17980	0.60	0.0202	Arabidopsis NAC domain containing protein 71, no apical meristem (NAM) family protein, putative transcription factor	ANAC071	
At5g05840	0.60	0.0013	unknown protein		
At4g26410	0.60	0.0171	unknown protein		
At3g49840	0.60	0.0213	proline-rich family protein		
At5g20270	0.60	0.0045	heptahelical transmembrane protein 1, unknown function	HHP1	Methyl jasmonate response
At5g58010	0.59	0.0445	basic helix-loop-helix (bHLH) family protein, putative transcription factor		
At5g35630	0.59	0.0251	glutamine synthetase 2	GS2	
At4g02090	0.59	0.0392	unknown protein		
At2g30620	0.58	0.0143	histone H1.2	H1.2	
At3g44990	0.58	0.0334	xyloglucan:xyloglucosyl transferase 8, putative xyloglucan endotransglycosylase, putative endo-xyloglucan transferase	XTR8	Cold-, drought- and UVB-stress response/ Methyl jasmonate response
At3g12820	0.58	0.0003	myb domain protein 10, myb family transcription factor	MYB10	Upregulated under iron deficiency
At3g51160	0.58	0.0117	GDP-D-mannose-4,6-dehydratase 2, first step in the de novo synthesis of GDP-L-fucose	MUR1	
At3g62280	0.57	0.0138	GDSL-motif lipase/hydrolase family protein		
At4g19680	0.57	0.0020	iron-responsive transporter 2, ZRT-, IRT-related protein, ZIP family iron transporter	IRT2	Upregulated under iron deficiency/ Metal homeostasis
At1g59960	0.57	0.0119	putative aldo/keto reductase		
At3g19010	0.56	0.0058	oxidoreductase, 2OG-Fe(II) oxygenase family protein		Methyl jasmonate response
At4g35720	0.55	0.0244	unknown protein		
At2g29250	0.55	0.0052	putative lectin protein kinase		
At1g27740	0.55	0.0006	basic helix-loop-helix (bHLH) family protein, putative transcription factor		
At5g60530	0.55	0.0468	late embryogenesis abundant protein-related / LEA protein-related		
At1g30220	0.55	0.0013	putative inositol transporter 2 sugar transporter family protein, major facilitator superfamily	INT2	
At2g15830	0.55	0.0204	unknown protein		
At2g19410	0.54	0.0002	protein kinase family protein, putative serine/threonin protein kinase		Upregulated under iron deficiency
At3g23730	0.54	0.0443	putative xyloglucan:xyloglucosyl transferase, putative xyloglucan endotransglycosylase, putative endo-xyloglucan transferase		
At2g14247	0.53	0.0051	unknown protein		
At2g41240	0.53	0.0397	basic helix-loop-helix (bHLH) family protein, putative transcription factor		
At5g22430	0.52	0.0191	unknown protein		

AGI Number	Signal ratio	*P* value	Annotation	Name	Class
At4g33666	0.52	0.0004	unknown protein		
At4g30120	0.52	0.0128	heavy metal ATPase 3, P1B-type (CPX) Cd/Zn/Pb ATPase	HMA3	Upregulated under iron deficiency/ Metal homeostasis
At1g69050	0.51	0.0282	unknown protein		
At5g15180	0.51	0.0332	peroxidase 56 precursor, putative peroxidase, haem peroxidase domain	PER56	
At4g33730	0.51	0.0094	putative pathogenesis-related protein		
At2g34910	0.50	0.0358	unknown protein		
At3g61410	0.50	0.0001	unknown protein, similar to protein kinase family protein, U-box domain-containing protein		Upregulated under iron deficiency
At5g63180	0.50	0.0024	pectate lyase family protein		NaCl response
At3g61930	0.48	0.0109	unknown protein		Upregulated under iron deficiency
At2g36100	0.48	0.0476	integral membrane family protein		
At3g58060	0.48	0.0039	metal tolerance protein 8, cation diffusion facilitator family of membrane transport proteins, putative Mn transporter	MTP8	Upregulated under iron deficiency/ Metal homeostasis
At2g28160	0.45	0.0011	Fe-deficiency induced transcription factor 1, basic helix-loop-helix (bHLH) family protein	FIT1/ bHLH29	Upregulated under iron deficiency/ Metal homeostasis/ OPDA response
At3g53460	0.42	0.0134	29 kDa ribonucleoprotein, chloroplast / RNA-binding protein cp 29	CP29	
At5g45105	0.41	0.0000	ZRT-, IRT-related protein 8, ZIP family putative zinc transporter	ZIP8	Metal homeostasis
At4g25790	0.39	0.0069	putative pathogenesis-related protein, allergen V5/Tpx-1-related family protein		
At5g57625	0.39	0.0278	putative pathogenesis-related protein, allergen V5/Tpx-1-related family protein		
At1g73120	0.36	0.0007	unknown protein		Upregulated under iron deficiency
At2g17500	0.36	0.0094	auxin efflux carrier family protein		Methyl jasmonate response
At3g12900	0.34	0.0000	oxidoreductase, 2OG-Fe(II) oxygenase family protein		Upregulated under iron deficiency
At5g02780	0.31	0.0000	In2-1-like protein, putative glutathione-S-transferase		Upregulated under iron deficiency
At1g01580	0.28	0.0131	ferric reduction oxidase 2, ferric-chelate reductase, responsible for the majority of iron(III)-chelate reduction at the root surface	FRO2	Upregulated under iron deficiency/ Metal homeostasis
At3g56980	0.28	0.0016	OBP3-responsive gene 3, basic helix-loop-helix (bHLH) family protein, putative transcription factor	bHLH039	Upregulated under iron deficiency/ Metal homeostasis
At3g60330	0.24	0.0099	Arabidopsis H+-ATPASE 7, plasma membrane P-type proton ATPase	AHA7	Upregulated under iron deficiency/ OPDA response
At3g58810	0.19	0.0012	metal tolerance protein 3, cation diffusion facilitator family of membrane transport proteins, vacuolar sequestration of Zn/Co	MTP3	Upregulated under iron deficiency/ metal homeostasis/ OPDA response
