# Supplementary material for: Wounding of Arabidopsis halleri leaves enhances cadmium accumulation that acts as a defense against herbivory
Source: Biometals. 2015 Mar 10;28(3):521–8. doi: 10.1007/s10534-015-9829-9 (PMC4427619; doi:10.1007/s10534-015-9829-9)
Supplement: Supplementary file 2 — Supplementary material 2 (PDF 29 kb) [file 10534_2015_9829_MOESM2_ESM.pdf]

Supplementary Table 2. Genes differentially expressed in roots of *A. thaliana* 5 h after leaf wounding. Given are AGI number, the ratio of the microarray signal of wounded *vs*. non-wounded plants calculated from the arithmetic mean of log-transformed values, annotation, short gene name, and functional or regulatory class for selected relevant classes. Microarray expression signals of shown genes changed by > 1.5-fold (increase or decrease) in response to leaf wounding, compared to non-wounded controls ($P < 0.05$). Plants were transfered into a hydroponic culture solution supplemented with 0.5 µM Cd 5 d prior to simulated herbivory on a single leaf. Annotations are underlined for genes differentially expressed in response to wounding when compared to controls in both *A. halleri* and in *A. thaliana*. OPDA: 12-oxo-phytodienoic acid.

AGI Number	Signal ratio	*P* value	Annotation	Name	Class
At3g23820	1.82	0.0083	UDP-D-Glucuronate 4-epimerase 6, similar to NAD-dependent epimerase/dehydratase family protein	GAE6	
At1g19180	1.81	0.0221	unknown protein		Local herbivory response/Cold-, drought- and UVB-stress response/ Methyl jasmonate response
At2g43680	1.80	0.0048	calmodulin-binding family protein	IQD14	
At4g11320	1.78	0.0356	cysteine proteinase similar to RD21A		Local herbivory response
At3g22440	1.73	0.0038	hydroxyproline-rich glycoprotein family protein		
At1g19570	1.73	0.0040	dehydroascorbate reductase 1	DHAR1	Local herbivory response/ Methyl jasmonate response
At4g33420	1.72	0.0043	peroxidase 47 precursor, putative peroxidase, haem peroxidase domain	PER47	Methyl jasmonate response
At3g47810	1.72	0.0167	calcineurin-like phosphoesterase family protein, putative serine/threonine phosphatase	MAG1	
At4g22010	1.70	0.0169	SKU5 similar 4, multi-copper oxidase type I family protein	SKS4	
At3g16450	1.69	0.0112	jacalin lectin family protein		
At2g30130	1.69	0.0057	phosphoenolpyruvate carboxykinase 1, lateral organ boundaries (LOB) domain protein 12 (LBD12)	ASL5/ LBD12	
At1g31730	1.69	0.0070	putative clathrin-binding epsilon-adaptin		
At2g27860	1.69	0.0065	UDP-D-apiose/UDP-D-xylose synthase 1	AXS1	
At1g52060	1.68	0.0078	jacalin lectin family protein		
At2g47180	1.65	0.0124	galactinol synthase 1	GOLS1	Local herbivory response/ Cold-, drought- and UVB-stress response/ NaCl response/ Methyl jasmonate response
At3g63460	1.64	0.0130	embryo defective 2221, WD-40 repeat family protein	AMB2221	
At4g14905	1.64	0.0006	kelch repeat-containing F-box family protein		
At5g60980	1.64	0.0046	nuclear transport factor 2 (NTF2) family protein / RNA recognition motif (RRM)-containing protein		
At3g49130	1.62	0.0064	RNA binding similar to SWAP (Suppressor-of-White-Apricot)/surp domain-containing protein		
At3g14860	1.62	0.0035	NHL repeat-containing protein		
At5g48385	1.61	0.0062	similar to hydroxyproline-rich glycoprotein family protein		
At1g29660	1.61	0.0078	GDSL-motif lipase/hydrolase family protein		NaCl response/ Methyl jasmonate response
At1g18040	1.61	0.0140	cyclin-dependent kinase D1;3	CDKD1;3	
At1g75800	1.58	0.0180	pathogenesis-related thaumatin family protein		Methyl jasmonate response
At4g32020	1.57	0.0120	unknown protein		Local herbivory response/ NaCl response
At4g34150	1.57	0.0148	C2 domain-containing protein		
At1g23960	1.56	0.0022	unknown protein		
At3g53100	1.56	0.0018	GDSL-motif lipase/hydrolase family protein		
At3g47470	1.56	0.0046	Photosystem I light harvesting complex gene 4, chlorophyll a/b binding protein	LHCA4	
At2g43520	1.55	0.0016	trypsin inhibitor protein 2, putative trypsin inhibitor	TI2	Methyl jasmonate response
At1g10840	1.55	0.0034	eukaryotic translation initiation factor 3 subunit H1, eIF-3 gamma	TIF3H1	
At4g15910	1.55	0.0027	drought-induced 21	DI21	
At3g15240	1.54	0.0190	unknown protein		
At4g11070	1.54	0.0180	WRKY DNA-binding protein 41, transcription factor	WRKY41	
At1g14710	1.54	0.0084	hydroxyproline-rich glycoprotein family protein, 2-OG-Fe(II) oxygenase family protein		
At3g01680	1.53	0.0078	unknown protein, putative thioredoxin domain		
At2g41370	1.52	0.0249	Blade on petiole 2, cytoplasmic and nuclear-localized NPR1 like protein with BTB/POZ domain and ankyrin repeats	BOP2	
At1g22335	1.52	0.0147	similar to RNA recognition motif (RRM)-containing protein		
At5g54770	1.52	0.0013	Thiazole requiring 1, thiamine biosynthesis and mitochondrial DNA damage tolerance	THI1/ ARA6	NaCl response

AGI Number	Signal ratio	*P* value	Annotation	Name	Class
At3g48650	1.52	0.0057	pseudogene, At14a-related protein		
At4g01950	1.51	0.0047	glycerol-3-phosphate acyl transferase 3	GPAT3	OPDA response/ NaCl response/ Methyl jasmonate response
At3g18540	1.51	0.0023	unknown protein		
At5g65790	1.51	0.0080	myb domain protein 68, transcription factor	MYB68	Methyl jasmonate response
At4g12650	1.50	0.0194	endomembrane protein 70, putative		
At3g24180	0.67	0.0222	unknown protein		
At3g13062	0.67	0.0079	unknown protein		
At3g54270	0.67	0.0043	sucrose-phosphatase 3	SPP3	
At1g07795	0.67	0.0205	unknown protein		
At2g45220	0.67	0.0351	pectinesterase family protein		
At5g46230	0.67	0.0106	unknown protein		
At5g02740	0.67	0.0050	unknown protein, nuleotide binding		NaCl response
At3g21700	0.66	0.0051	unknown protein, similar to GTP-binding family protein, similar to Rab-type Ras small GTPase		
At4g29220	0.66	0.0325	phosphofructokinase family protein		Upregulated under iron deficiency
At3g07780	0.66	0.0053	unknown protein, zinc binding		
At1g74055	0.66	0.0087	unknown protein		
At5g57360	0.66	0.0217	ubiquitin-protein ligase, clock-associated PAS protein	ZTL	NaCl response
At5g13150	0.66	0.0103	exocyst subunit EXO70 family protein C1	EXO70C1	
At5g19700	0.66	0.0284	MATE efflux protein-related		
At5g65683	0.66	0.0023	zinc finger (C3HC4-type RING finger) family protein, ubiquitin-protein ligase		
At5g50660	0.66	0.0359	unknown protein		
At3g51990	0.66	0.0048	protein kinase family protein		
At1g02900	0.66	0.0089	Rapid Alkalinization Factor (RALF)-like 1 protein	RALFL1	
At3g12870	0.66	0.0009	unknown protein		
At3g51930	0.66	0.0022	WD-40 repeat family protein, transducin family protein		
At2g40320	0.66	0.0210	unknown protein, similar to steroid hormone receptor/ transcription factor		
At2g31120	0.66	0.0074	unknown protein		
At5g40730	0.66	0.0329	arabinogalactan protein 24	AGP24	
At1g27640	0.66	0.0196	unknown protein		
At4g32950	0.66	0.0333	putative protein phosphatase 2c		
At2g29250	0.66	0.0407	lectin protein kinase, putative		
At1g79410	0.66	0.0251	organic cation transporter-related, major facilitator superfamily		Methyl jasmonate reponse
At2g36100	0.66	0.0351	integral membrane family protein		
At5g19970	0.66	0.0222	unknown protein		Methyl jasmonate response
At3g61060	0.66	0.0123	Phloem protein 2-A13, F-box family protein, galactose-binding like	PP2-A13	Methyl jasmonate response
At4g13510	0.66	0.0123	ammonium transporter 1;1, high-affinity plasma membrane ammonium uptake system	AMT1;1	Methyl jasmonate-response
At3g16170	0.66	0.0440	acyl-activating enzyme 13	AAE13	
At1g72160	0.66	0.0278	patellin-3, SEC14 cytosolic factor family protein / phosphoglyceride transfer family protein	PATL3	
At3g50740	0.66	0.0083	UDP-glucosyl transferase 72E1	UGT72E1	Upregulated under iron deficiency
At2g17705	0.66	0.0038	putative protein-methionine-S-oxide reductase		
At5g54585	0.66	0.0326	unknown protein		
At4g20320	0.66	0.0085	putative CTP synthase / putative UTP-ammonia ligase		
At3g13910	0.65	0.0127	unknown protein		Methyl jasmonate response
At1g65690	0.65	0.0490	harpin-induced protein-related / HIN1-related / harpin-responsive protein-related		OPDA response/ Methyl jasmonate response
At4g00650	0.65	0.0020	FRIGIDA protein	FRI	
At2g24710	0.65	0.0057	glutamate receptor 2.3, putative ligand-gated ion channel subunit	GLR2.3	
At3g51160	0.65	0.0108	GDP-D-mannose-4,6-dehydratase 2, first step in the de novo synthesis of GDP-L-fucose	MUR1	
At2g29870	0.65	0.0462	major intrinsic protein (MIP) family protein		
At3g59840	0.65	0.0070	unknown protein		NaCl response
At5g37540	0.65	0.0189	aspartyl protease family protein		
At5g14730	0.65	0.0039	unknown protein		Methyl jasmonate response
At5g65160	0.65	0.0094	tetratricopeptide repeat (TPR)-containing protein		
At4g33260	0.65	0.0044	WD-40 repeat family protein	CDC20.2	
At2g44390	0.65	0.0403	DC1 domain-containing protein		
At1g29640	0.65	0.0135	unknown protein		
At3g12500	0.65	0.0171	basic endochitinase	CHIB	
At1g65490	0.65	0.0118	unknown protein		Methyl jasmonate response

AGI Number	Signal ratio	*P* value	Annotation	Name	Class
At3g04530	0.65	0.0072	phosphoenolpyruvate carboxylase kinase 2	PPCK2	
At1g02120	0.65	0.0126	Vascular-associated death 1, GRAM domain-containing protein	VAD1	
At4g37520	0.65	0.0084	peroxidase 50, putative peroxidase, haem peroxidase domain	PER50	
At2g34500	0.65	0.0087	cytochrome P450, family 710, subfamily A, polypeptide 1, C-22 sterol desaturase/ oxygen binding	CYP710A1	
At1g74770	0.65	0.0189	unknown protein		
At5g02420	0.64	0.0135	unknown protein		
At3g48360	0.64	0.0042	BTB and TAZ domain protein 2, transcriptional regulator of the TAC1-mediated telomerase activation pathway	BT2	Cold-, drought- and UVB-stress response
At5g58270	0.64	0.0044	STARIK 1, mitochondrial half-size ABC transporter of ATM subfamily	STA1	
At1g76680	0.64	0.0072	12-oxophytodienoate reductase 1, jasmonic acid biosynthesis	OPR1	Local herbivory response
At3g01290	0.64	0.0162	band 7 family protein		
At4g22070	0.64	0.0039	WRKY DNA-binding protein 31, transcription factor	WRKY31	
At3g11520	0.64	0.0012	cyclin B1;3, cyclin-dependent protein kinase regulator, B-type mitotic cyclin	CYCB1;3	
At4g23870	0.64	0.0005	unknown protein		
At2g18890	0.64	0.0162	protein kinase family protein, serine/threonine kinase domain		
At3g60330	0.64	0.0316	Arabidopsis H+-ATPASE 7, plasma membrane P-type proton ATPase	AHA7	Upregulated under iron deficiency/ OPDA response
At3g23730	0.64	0.0033	putative xyloglucan:xyloglucosyl transferase, putative xyloglucan endotransglycosylase, putative endo-xyloglucan transferase		
At4g32650	0.64	0.0410	putative inward rectifying potassium channel	ATKC1/ KAT3/ AKT4	Methyl jasmonate response
At2g34000	0.64	0.0470	zinc finger protein-related		
At5g65250	0.64	0.0102	unknown protein		
At1g72200	0.64	0.0026	zinc finger (C3HC4-type RING finger) family protein	ATL1N	Methyl jasmonate response
At1g55420	0.64	0.0314	embryo sac development arrest 11, DC1 domain-containing protein	EDA11	
At3g16920	0.63	0.0059	glycoside hydrolase family 19 protein, chitinase-like		
At2g34790	0.63	0.0161	embryo sac development arrest 28, maternal effect embryo arrest 23, FAD-binding domain-containing protein	EDA28/ MEE23	
At1g78020	0.63	0.0125	senescence-associated protein-related		
At1g55850	0.63	0.0119	Cellulose synthase-like E1	CSLE1	OPDA response
At5g37130	0.63	0.0072	tetratricopeptide repeat (TPR)-containing protein		
At1g74940	0.63	0.0080	senescence-associated protein-related		NaCl response
At4g37010	0.63	0.0356	putative caltractin/ putative centrin		Methyl jasmonate response
At4g38340	0.63	0.0095	RWP-RK domain-containing protein		
At5g55970	0.63	0.0337	zinc finger (C3HC4-type RING finger) family protein		
At3g13950	0.63	0.0259	unknown protein		
At1g66200	0.63	0.0361	glutamine synthase clone R2, glutamate-ammonia ligase encodes a cytosolic glutamate synthetase	GSR2	
At2g23960	0.63	0.0105	putative defense-related protein		
At5g20820	0.63	0.0066	auxin-responsive protein-related		
At5g35630	0.63	0.0118	glutamine synthetase 2, glutamate-ammonia ligase chloroplastic glutamine synthetase	GS2	
At2g19060	0.63	0.0199	GDSL-motif lipase/hydrolase family protein		
At1g63570	0.63	0.0188	receptor-like protein kinase-related		
At1g51790	0.63	0.0375	putative leucine-rich repeat protein kinase		
At3g14840	0.62	0.0177	leucine-rich repeat family protein / protein kinase family protein		
At3g08040	0.62	0.0081	ferric reductase defective 3, MATE efflux family protein, plasma membrane citrate effluxer	FRD3	Metal homeostasis
At4g26540	0.62	0.0480	protein kinase family protein, similar to leucine-rich repeat transmembrane protein kinase		
At1g73430	0.62	0.0074	sec34-like family protein		
At4g26960	0.62	0.0003	unknown protein		

AGI Number	Signal ratio	*P* value	Annotation	Name	Class
At4g33730	0.62	0.0037	putative pathogenesis-related protein		
At1g08090	0.62	0.0481	nitrate transporter 2.1, high-affinity plasma membrane nitrate uptake system, repressor of lateral root initiation	NRT2;1	
At5g55710	0.62	0.0101	unknown protein		
At2g13810	0.62	0.0068	AGD2-like defense response protein 1, transaminase	ALD1	
At1g76700	0.62	0.0030	DNAJ heat shock N-terminal domain-containing protein		
At4g18510	0.62	0.0112	Clavata 3-/ ESR-related 2	CLE2	
At2g26250	0.62	0.0053	beta-ketoacyl-CoA synthase family (FIDDLEHEAD) (FDH)	FDH	
At2g37750	0.62	0.0164	unknown protein		OPDA response/ Methyl jasmonate response
At4g25790	0.62	0.0069	putative pathogenesis-related protein, allergen V5/Tpx-1-related family protein		
At4g15550	0.62	0.0228	UDP-glucose:indole-3-acetate beta-D-glucosyltransferase, indole-3-acetate beta-D-glucosyl transferase	IAGLU	
At3g10080	0.61	0.0049	putative germin-like protein, manganese ion binding, nutrient reservoir		
At1g63245	0.61	0.0094	Clavata 3-/ ESR-related 14	CLE14	
At3g62780	0.61	0.0051	C2 domain-containing protein		
At1g03660	0.61	0.0418	unknown protein, similar to ankyrin repeat family protein		
At1g63450	0.61	0.0301	exostosin family protein		
At5g06570	0.61	0.0099	unknown protein, similar to CXE carboxylesterase		
At1g09350	0.61	0.0194	galactinol synthase 3	GOLS3	
At4g17490	0.60	0.0231	ethylene-responsive element-binding factor 6	ERF6	Cold-, drought- and UVB-stress response
At5g64120	0.60	0.0067	peroxidase 71, putative peroxidase, haem peroxidase domain	PER71	
At5g62860	0.60	0.0070	F-box family protein-related		
At3g60420	0.60	0.0186	unknown protein, PRIB5 domain, phosphoglycerate mutase domain		OPDA response
At5g01810	0.60	0.0115	CBL-interacting protein kinase 15, serine/threonine protein kinase	CIPK15	Methyl jasmonate response
At5g44130	0.60	0.0243	fasciclin-like arabinogalactan-protein	FLA13	Methyl jasmonate response
At2g19990	0.60	0.0462	pathogenesis-related protein 1	PR1	
At4g08810	0.60	0.0028	short under blue light 1, calcium-binding protein involved in cryptochrome and phytochrome coaction	SUB1	
At4g10270	0.60	0.0218	wound-responsive family protein		NaCl response
At2g15340	0.60	0.0031	glycine-rich protein		
At1g02930	0.59	0.0127	glutathione S-transferase	GSTF6	Local herbivory response
At3g19030	0.59	0.0137	unknown protein		Cold-, drought- and UVB-stress response/ NaCl response
At3g46190	0.59	0.0339	meprin and TRAF homology domain-containing protein / MATH domain-containing protein		
At5g63160	0.59	0.0091	BTB and TAZ domain protein 1, transcription regulator	BT1	
At2g22800	0.59	0.0013	homeobox-leucine zipper protein 9, transcription factor	HAT9	
At5g05440	0.59	0.0066	unknown protein		Methyl jasmonate response
At1g47480	0.58	0.0370	unknown protein, similar to CXE carboxylesterase		
At5g65925	0.58	0.0035	unknown protein		
At3g49840	0.58	0.0033	proline-rich family protein		
At2g27370	0.58	0.0107	integral membrane family protein		
At1g08420	0.58	0.0266	kelch repeat-containing protein/ serine/threonine phosphoesterase family protein		
At1g19390	0.58	0.0211	wall-associated receptor kinase-like 11	WAKL11	NaCl response
At5g47200	0.58	0.0039	Arabidopsis Rab GTPase homolog D2b, GTP-binding protein	RABD2b/ Rab1A	
At4g26080	0.58	0.0017	ABA insensitive 1, protein phosphatase 2C	ABI1	Local herbivory response/ NaCl response/ Methyl jasmonate response
At1g33880	0.58	0.0468	avirulence-responsive family protein / avirulence induced gene (AIG1) family protein		
At3g54420	0.58	0.0225	class IV chitinase	EP3	Local herbivory response
At5g15120	0.58	0.0020	unknown protein		
At3g52420	0.57	0.0270	putative outer envelope membrane protein		
At5g45105	0.57	0.0485	ZRT-, IRT-related protein 8, ZIP family putative zinc transporter	ZIP8	Metal homeostasis

AGI Number	Signal ratio	*P* value	Annotation	Name	Class
At5g06490	0.57	0.0111	zinc finger (C3HC4-type RING finger) family protein	L5D	Upregulated under iron deficiency
At2g27550	0.57	0.0055	centroradialis (CEN), phosphatidylethanolamine binding	ATC	NaCl response
At1g03080	0.57	0.0022	kinase interacting family protein		Methyl jasmonate response
At3g19010	0.57	0.0165	oxidoreductase, 2OG-Fe(II) oxygenase family protein		Methyl jasmonate response
At4g21680	0.56	0.0049	proton-dependent oligopeptide transport (POT) family protein		Upregulated under iron deficiency/ NaCl response/ Methyl jasmonate response
At5g59680	0.56	0.0330	leucine-rich repeat protein kinase, putative		
At1g78000	0.56	0.0324	sulfate transporter 1;2, high-affinity plasma membrane sulfate uptake system	SULTR1;2	OPDA response
At2g35850	0.56	0.0020	unknown protein		
At1g62510	0.56	0.0222	protease inhibitor/seed storage/lipid transfer protein (LTP) family protein		NaCl response
At3g46900	0.56	0.0052	Copper transporter 2, plasma membrane copper uptake system	COPT2	Upregulated under iron deficiency/Metal homeostasis
At2g35000	0.56	0.0132	zinc finger (C3HC4-type RING finger) family protein		
At5g17820	0.55	0.0446	peroxidase 57, putative peroxidase, haem peroxidase domain	PER57	
At2g20670	0.55	0.0002	unknown protein		Methyl jasmonate response
At2g15830	0.55	0.0338	unknown protein		
At5g02780	0.54	0.0142	In2-1-like protein, putative glutathione-S-transferase		Upregulated under iron deficiency
At1g09090	0.54	0.0082	respiratory burst oxidase protein B (RbohB) / NADPH oxidase	RBOHB	Methyl jasmonate response
At2g39520	0.54	0.0321	unknown protein		
At4g09100	0.54	0.0105	zinc finger (C3HC4-type RING finger) family protein		
At1g59960	0.54	0.0050	putative aldo/keto reductase		
At5g60660	0.54	0.0050	plasma membrane intrinsic protein 2;4, water channel, major intrinsic protein (MIP) family	PIP2;4/ PIP2F	
At1g67810	0.53	0.0003	Fe-S metabolism associated domain-containing protein		OPDA response
At2g41970	0.53	0.0109	protein kinase, putative		
At2g20960	0.53	0.0289	unknown protein, phospholipase-like domain	pEARLI4	
At5g38910	0.53	0.0209	germin-like protein, putative		
At3g21710	0.53	0.0004	unknown protein		
At4g34930	0.52	0.0069	1-phosphatidylinositol phosphodiesterase-related		
At1g10170	0.52	0.0082	NF-X1 type zinc finger family protein		
At3g18170	0.52	0.0211	hypothetical protein		
At2g34180	0.52	0.0016	CBL-interacting protein kinase 13	CIPK13	
At4g08620	0.51	0.0243	sulfate transporter 1;1, high affinity plasma membrane sulfate uptake system	SULTR1;1	
At3g07720	0.51	0.0085	kelch repeat-containing protein		Upregulated under iron deficiency
At1g60750	0.51	0.0013	pseudogene, aldo/keto reductase family		OPDA response
At3g13650	0.51	0.0056	disease resistance response		OPDA response
At3g63360	0.50	0.0329	defensin-like (DEFL) family protein		
At5g57625	0.48	0.0458	putative pathogenesis-related protein, allergen V5/Tpx-1-related family protein		
At5g65980	0.48	0.0068	auxin efflux carrier family protein		
At5g66985	0.47	0.0091	unknown protein		
At5g45380	0.47	0.0070	sodium:solute symporter family protein		
At1g76800	0.44	0.0060	putative nodulin	CCCl4	Metal homeostasis
At3g58810	0.43	0.0077	metal tolerance protein 3, cation diffusion facilitator family of membrane transport proteins, vacuolar sequestration of Zn/Co	MTP3	Upregulated under iron deficiency/ Metal homeostasis/OPDA response
At1g73120	0.43	0.0068	unknown protein		Upregulated under iron deficiency
At4g33070	0.42	0.0004	putative pyruvate decarboxylase		Methyl jasmonate response
At1g01580	0.40	0.0187	ferric reduction oxidase 2, ferric-chelate reductase, responsible for the majority of iron(III)-chelate reduction at the root surface	FRO2	Upregulated under iron deficiency/ Metal homeostasis
