# Supplementary material for: Wounding of Arabidopsis halleri leaves enhances cadmium accumulation that acts as a defense against herbivory
Source: Biometals. 2015 Mar 10;28(3):521–8. doi: 10.1007/s10534-015-9829-9 (PMC4427619; doi:10.1007/s10534-015-9829-9)
Supplement: Supplementary file 3 — Supplementary material 3 (PDF 33 kb) [file 10534_2015_9829_MOESM3_ESM.pdf]

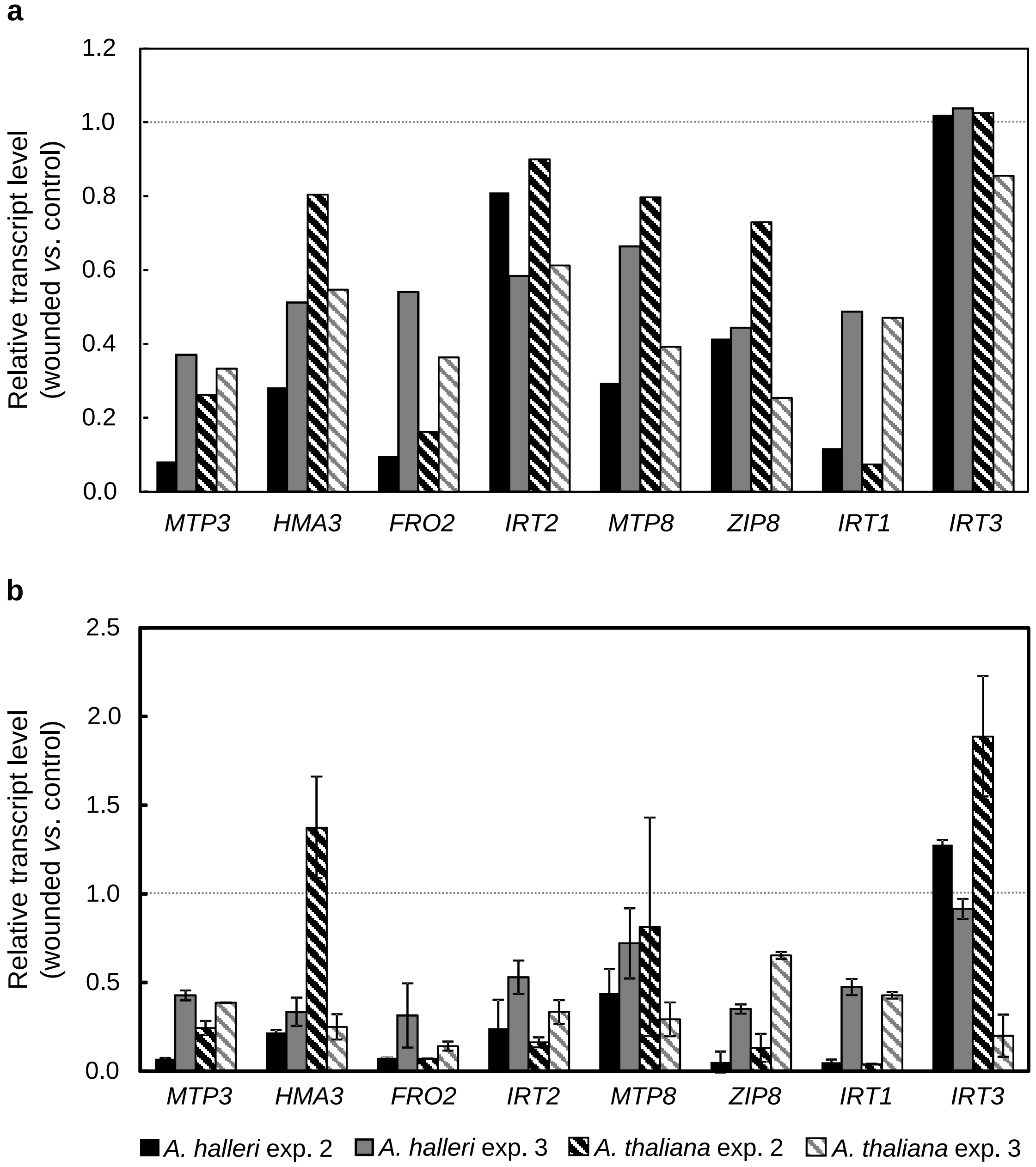

Supplementary Figure 1: Real-time RT-PCR (qPCR) validation of gene expression determined by microarray hybridization. For seven selected iron deficiency response metal homeostasis genes (*MTP3*, *HMA3*, *FRO2*, *IRT2*, *MTP8*, *ZIP8*, *IRT1*) and one Zn-deficiency response metal homeostasis gene as a negative control (IRT3), bars show transcript levels in leaves of wounded relative to non-wounded *A. halleri* and *A. thaliana* plants based on **(a)** CATMA microarray hybridization and **(b)** quantitative real-time RT-PCR, using the same RNA. For qPCR, values represent fold changes in gene expression calculated by dividing relative transcript levels (RTL) of wounding treatments by RTL of controls (see Talke *et al.*, 2006). Across all four replicates, average microarray values for *IRT1* (*A.h.* fold change: 0.44, *P* = 0.073, *A.t.* fold change: 0.39, *P* = 0.069) did not pass the stringent cut-off values. According to the microarray data, *IRT3* transcript levels showed no wounding-dependent regulation and served as a negative control (*A.h.* fold change: 1.02, *P* = 0.631, *A.t.* fold change: 1.05, *P* = 0.482). *A.h., A. halleri*; *A.t., A. thaliana*.
